# Upregulation of *SLC2A3* gene and prognosis in colorectal carcinoma: analysis of TCGA data

**DOI:** 10.1186/s12885-019-5475-x

**Published:** 2019-04-03

**Authors:** Eunyoung Kim, Sohee Jung, Won Seo Park, Joon-Hyop Lee, Rumi Shin, Seung Chul Heo, Eun Kyung Choe, Jae Hyun Lee, Kwangsoo Kim, Young Jun Chai

**Affiliations:** 1 Department of Surgery, National Medical Center, Seoul, Republic of Korea; 2 Division of Clinical Bioinformatics, Biomedical Research Institute, Seoul National University Hospital, Seoul, Republic of Korea; 3 Department of Surgery, Graduate College of Medicine, Kyung Hee University, Seoul, Republic of Korea; 4 Department of Surgery, Gachon University Gil Medical Center, Incheon, Republic of Korea; 5 Department of Surgery, Seoul Metropolitan Government - Seoul National University Boramae Medical Center, 20 Boramae-ro 5-gil, Dongjak-gu, Seoul, 156-70 Republic of Korea; 6 Department of Surgery, Seoul National University Hospital Healthcare System, Gangnam Center, Seoul, Republic of Korea; 7 Department of Statistics, Korea University, Seoul, Republic of Korea

**Keywords:** Colorectal cancer, Solute carrier 2A (*SLC2A*), Glucose transporter (GLUT), The cancer genome atlas (TCGA), Prognosis

## Abstract

**Background:**

Upregulation of *SLC2A* genes that encode glucose transporter (GLUT) protein is associated with poor prognosis in many cancers. In colorectal cancer, studies reporting the association between overexpression of GLUT and poor clinical outcomes were flawed by small sample sizes or subjective interpretation of immunohistochemical staining. Here, we analyzed mRNA expressions in all 14 *SLC2A* genes and evaluated the association with prognosis in colorectal cancer using data from the Cancer Genome Atlas (TCGA) database.

**Methods:**

In the present study, we analyzed the expression of *SLC2A* genes in colorectal cancer and their association with prognosis using data obtained from the TCGA for the discovery sample, and a dataset from the Gene Expression Omnibus for the validation sample.

**Results:**

*SLC2A3* was significantly associated with overall survival (OS) and disease-free survival (DFS) in both the discovery sample (345 patients) and validation sample (501 patients). High *SLC2A3* expression resulted in shorter OS and DFS. In multivariate analyses, high *SLC2A3* levels predicted unfavorable OS (adjusted HR 1.95, 95% CI 1.22–3.11; *P* = 0.005) and were associated with poor DFS (adjusted HR 1.85, 95% CI 1.10–3.12; *P* = 0.02). Similar results were found in the discovery set.

**Conclusion:**

Upregulation of the *SLC2A3* genes is associated with decreased OS and DFS in colorectal cancer patients. Therefore, assessment of *SLC2A3* gene expression may useful for predicting prognosis in these patients.

**Electronic supplementary material:**

The online version of this article (10.1186/s12885-019-5475-x) contains supplementary material, which is available to authorized users.

## Background

Colorectal cancer (CRC) is the third most common cancer and the fourth-leading cause of cancer death in the world [1, 2]. Most CRCs originate from non-cancerous lesions by one or a combination of three different mechanisms: chromosomal instability, CpG island methylator phenotype, and microsatellite instability [3]. Biomarkers of these cytogenetic alterations are of interest for diagnosis, prognostication, and anticancer drug development targeting CRC [4]. Despite research efforts, genetic biomarkers currently have limited value as diagnostic or prognostic markers [5].

Among the biomarkers, the solute carrier 2A (*SLC2A*) gene family that encodes glucose transporter (GLUT) proteins has been widely investigated. GLUT proteins facilitate glucose influx into cancer cells which is necessary for cancer cell proliferation. Upregulation of *SLC2A* genes is associated with poor prognosis in many cancers, including hepatocellular carcinoma, non–small cell lung cancer, and thyroid carcinoma [6–8].

An association between overexpression of the subtypes of GLUT proteins and poor clinical outcomes has been reported in CRC [9]. However, these studies were flawed by small sample sizes or subjective interpretation of immunohistochemical (IHC) staining. In this study, we analyzed the mRNA expression of all 14 *SLC2A* genes (corresponding to 14 GLUT proteins) and evaluated the associations with prognosis in CRC using data from the Cancer Genome Atlas (TCGA) database.

## Methods

### Data acquisition

The TCGA CRC data was downloaded from cBioPortal for Cancer Genomics (http://www.cbioportal.org/). The dataset contains survival data with clinical information, somatic mutations, and mRNA expression counts. For validation, we obtained independent microarray datasets (GSE39582) from the Gene Expression Omnibus (GEO). mRNA counts of the validation set were measured by the Affymetrix Human Genome U133 Plus 2.0 Array in a log2 scale. Gene expression of the discovery set was measured by Illumina HiSeq platform and transformed into log2 scale. According to the publication guidelines, the datasets may be used for publication without restriction or limitation (https://cancergenome.nih.gov/publications/publicationguidelines, https://www.ncbi.nlm.nih.gov/geo/info/disclaimer.html).

### Statistical analysis

The putative associations between conventional clinical pathology parameters (age at diagnosis, sex, American Joint Committee on Cancer (AJCC) 7th edition TNM stage, microsatellite instability, and mutational status of *KRAS* (v-Ki-ras2 Kirsten rat sarcoma viral oncogene homolog) and *BRAF* (v-Raf murine sarcoma viral oncogene homolog B1 genes) and survival outcome were assessed by Chi-square tests. The Cox proportional-hazards model was used to identify genes associated with survival and to estimate mortality hazard ratios (HRs). The optimal cut-off points for *SLC2A3* expression used to divide patients into low-risk and high-risk groups were determined using the MaxStat package of R software (Maximally selected Rank Statistics). Maxstat computes the maximally selected log-rank statistic to identify the cutpoint which provides the best separation (in which the standardized statistics take their maximum) into two groups. Kaplan-Meier analysis was performed to estimate the survival curves of the different subgroups and the log-rank test *(*Mantel–Cox) was used to compare the curve. Statistical analyses were performed using R statistical software (version 3.4.1) [10]. All *P*-value were two sided. A P-value less than 0.05 was considered statistically significant.

## Results

### Patient demographics

Our study sample comprised 846 patients. Patient characteristics of the discovery and validation set are shown in Table 1. The TCGA sample of 345 patients were the discovery set, and the GSE39582 sample of 501 patients were the validation set.

**Table 1:** Patient demographics

	Discovery Set (TCGA, *N* = 345)	Validation Set (GSE39582, *N* = 501)
Age, yr		
Median	67	68.1
Interquartile range	(55–75)	(59–76)
Sex		
Female	157 (46%)	229 (46%)
Male	188 (54%)	272 (54%)
AJCC TNM Stage		
I	54 (16%)	31 (6%)
II	130 (38%)	244 (49%)
III	111 (32%)	166 (33%)
IV	50 (14%)	60 (12%)
Microsatellite instability		
MSI-L and MSS	291 (84%)	
MSI-H	54 (16%)	
*BRAF* status		
V600E	50 (14%)	49 (10%)
Wild-type	295 (86%)	452 (90%)
*KRAS* status		
Mutant	144 (42%)	198 (40%)
Wild-type	201 (58%)	303 (60%)
OS event		
Event	78 (23%)	171 (34%)
Non-event	267 (77%)	330 (66%)
OS months		
Median	31.4	56.5
Range	(0–147.9)	(0–201)
DFS event		
Event	83 (24%)	150 (30%)
Non-event	222 (64%)	346 (69%)
Not available	40 (12%)	5 (1%)
DFS months		
Median	21	44
Range	(0–148)	(0–201)

### Association between clinical parameters and survival outcome

The associations between clinical variables and OS and DFS in the discovery set are summarized in Table 2. Age > 65 was associated with worse OS compared to age ≤ 65 (*P* < 0.001). TNM stage III and IV was associated with OS (P < 0.001) and DFS (P < 0.001). Other clinicopathological factors (sex, MSI, *BRAF*, or *KRAS* status) were not associated with OS or DFS.

**Table 2:** Association between the clinicopathological characteristics and survival outcome

	Overall survival			Disease-free survival		
	Non-event	Event	*P* value^a^	Non-event	Event	*P* value^a^
Age						
< = 65	141	23	< 0.001	115	43	1
> 65	126	55		107	40	
Sex						
Female	124	33	0.606	103	34	0.472
Male	143	45		119	49	
AJCC TNM stage						
I and II	157	27	< 0.001	137	32	< 0.001
III and IV	110	51		85	51	
MSI						
MSI-L and MSS	224	67	0.802	184	72	0.520
MSI-H	43	11		38	11	
*BRAF* status						
Wild-type	230	65	0.662	188	74	0.416
Mutant	37	13		34	9	
*KRAS* status						
Wild-type	154	47	0.783	133	43	0.252
Mutant	113	31		89	40	

^a^Calculated using the Chi-square test

### Prognostic value of SLC2A3

Table 3 displays the associations between the mRNA expression values of SLC2 family genes and survival outcomes. *SLC2A3* was significantly associated with both OS (*P* = 0.013) and DFS (*P* = 0.014). There were associations between the expression of *SLC2A6* and *SLC2A7* with worse OS (*P* = 0.048 and 0.019); and *SLC2A1* with worse DFS (*P* = 0.018).

**Table 3:** Univariate cox regression analysis of SLC2 family genes for OS and DFS

Gene^a^	OS		DFS	
	HR (95% CI)	*P* value^b^	HR (95% CI)	*P* value^b^
*SLC2A1*	1.13 (0.91–1.39)	0.271	1.28 (1.04–1.57)	0.018
*SLC2A2*	0.82 (0.61–1.09)	0.169	0.99 (0.8–1.24)	0.957
*SLC2A3*	1.33 (1.06–1.66)	0.013	1.32 (1.06–1.64)	0.014
*SLC2A4*	1.12 (0.9–1.39)	0.302	1.02 (0.82–1.27)	0.864
*SLC2A5*	1.17 (0.94–1.47)	0.159	1.04 (0.84–1.29)	0.703
*SLC2A6*	1.24 (1–1.54)	0.048	1.07 (0.87–1.32)	0.503
*SLC2A7*	1.23 (1.03–1.46)	0.019	1.06 (0.87–1.29)	0.562
*SLC2A8*	0.82 (0.67–1.01)	0.066	0.95 (0.78–1.17)	0.646
*SLC2A9*	0.86 (0.7–1.05)	0.135	1.00 (0.82–1.23)	0.965
*SLC2A10*	0.94 (0.76–1.17)	0.597	1.02 (0.82–1.27)	0.876
*SLC2A11*	1.14 (0.9–1.43)	0.277	1.11 (0.88–1.4)	0.369
*SLC2A12*	1.05 (0.84–1.32)	0.643	1.06 (0.86–1.32)	0.574
*SLC2A13*	0.89 (0.71–1.11)	0.305	1.02 (0.82–1.27)	0.835
*SLC2A14*	1.19 (0.96–1.49)	0.115	1.18 (0.96–1.46)	0.123

Abbreviations: HR, hazard ratio; CI, confidence interval

^a^Unit of measure is log2 of gene expression intensity

^b^Calculated using the Wald test

**Fig. 1:**
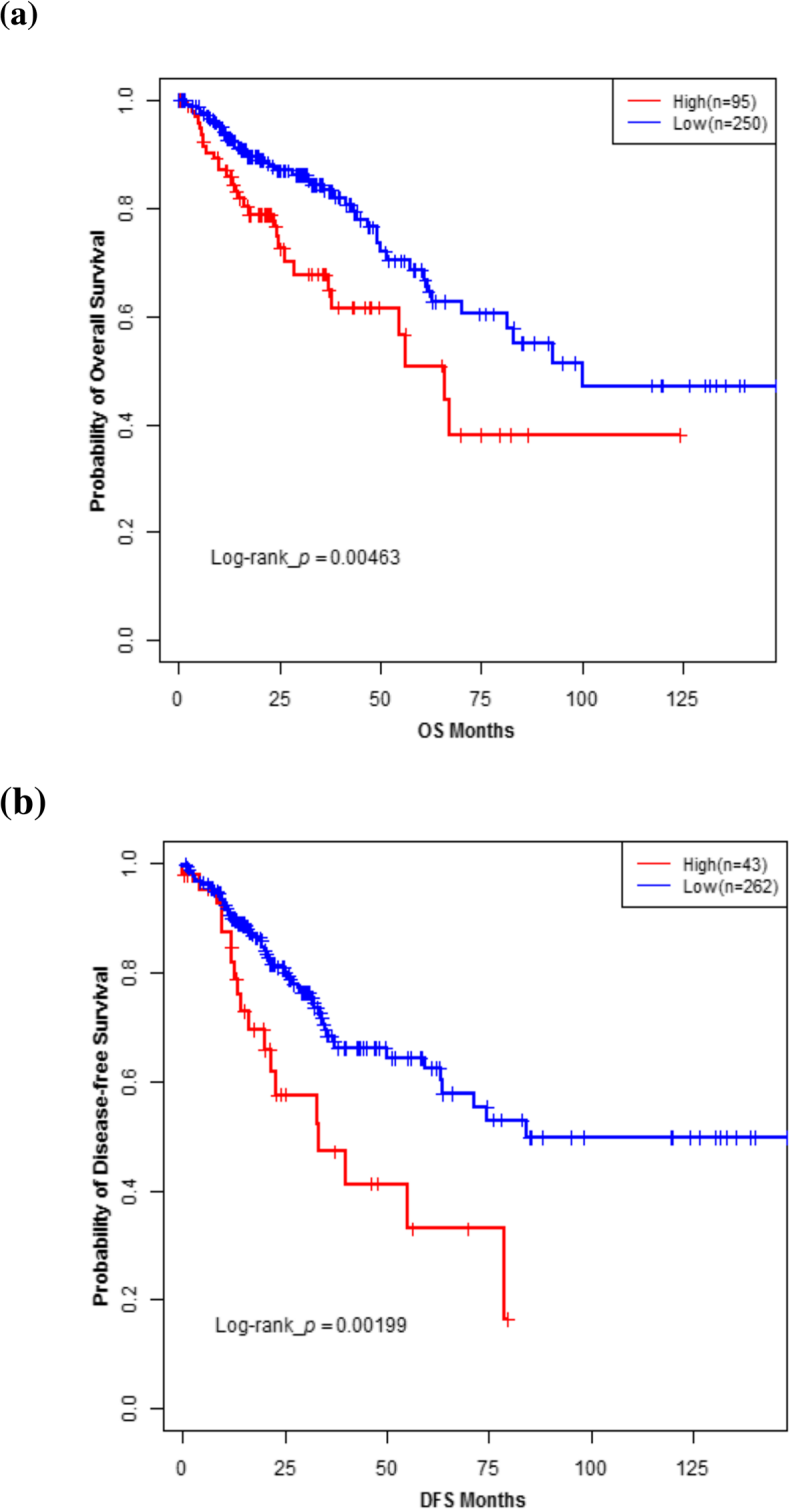
Kaplan-Meier survival analysis of colorectal cancer patients stratified by *SLC2A3* expression levels (Discovery set). Overall survival (**a**) and disease-free survival (**b**) curve of patients in discovery set with high versus low *SLC2A3* expression levels. *P*-values for significance of difference between high and low expression were calculated using the log-rank test

Patients were categorized into high and low *SLC2A3* expression groups according to the cut-off value determined by Maxstat method. The high expression group had worse OS (*P* = 0.005) and DFS (*P* = 0.002) compared to the low expression group (Fig. 1).

We explored the association between *SLC2A3* expression and survival outcome in a multivariate context (Table 4). In multivariate Cox regression analysis, after adjusting for clinical factors that were significantly associated with survival outcome (OS: age > 65, AJCC stage III and IV, DFS; AJCC stage III and IV), *SLC2A3* remained an independent poor prognostic factor for OS and DFS. *SLC2A3* level is associated with shorter OS with an adjusted hazard ratio of 1.98 (95% CI: 1.24–3.17; *P* = 0.004) and with poor disease-free survival (adjusted HR: 1.85, 95% CI: 1.10–3.12; *P* = 0.020).

**Table 4:** Univariate and multivariate Cox regression analyses of *SLC2A3* expression and the clinicopathological factors in the discovery set (TCGA COADREAD cohort)

	Overall survival		Disease-free survival	
	HR(95% CI of HR)	*P* value^a^	HR(95% CI of HR)	*P* value^a^
*SLC2A3* low	*1.00 (Reference)*		*1.00 (Reference)*	
UVA *SLC2A3* high	1.93 (1.21–3.07)	0.005	2.20 (1.32–3.68)	0.003
MVA *SLC2A3* high	1.98 (1.24–3.17)	0.004	1.85 (1.10–3.12)	0.020
Age < = 65	*1.00 (Reference)*			
Age > 65	2.61 (1.59–4.28)	< 0.001		
AJCC stage I, II	*1.00 (Reference)*		*1.00 (Reference)*	
AJCC stage III, IV	3.17 (1.96–5.11)	< 0.001	2.48 (1.58–3.89)	< 0.001

Abbreviations: *HR* hazard ratio, *CI* confidence interval, *UVA* univariate, *MVA* multivariate

^a^Calculated using the Wald test

### Validation set analysis

**Fig. 2:**
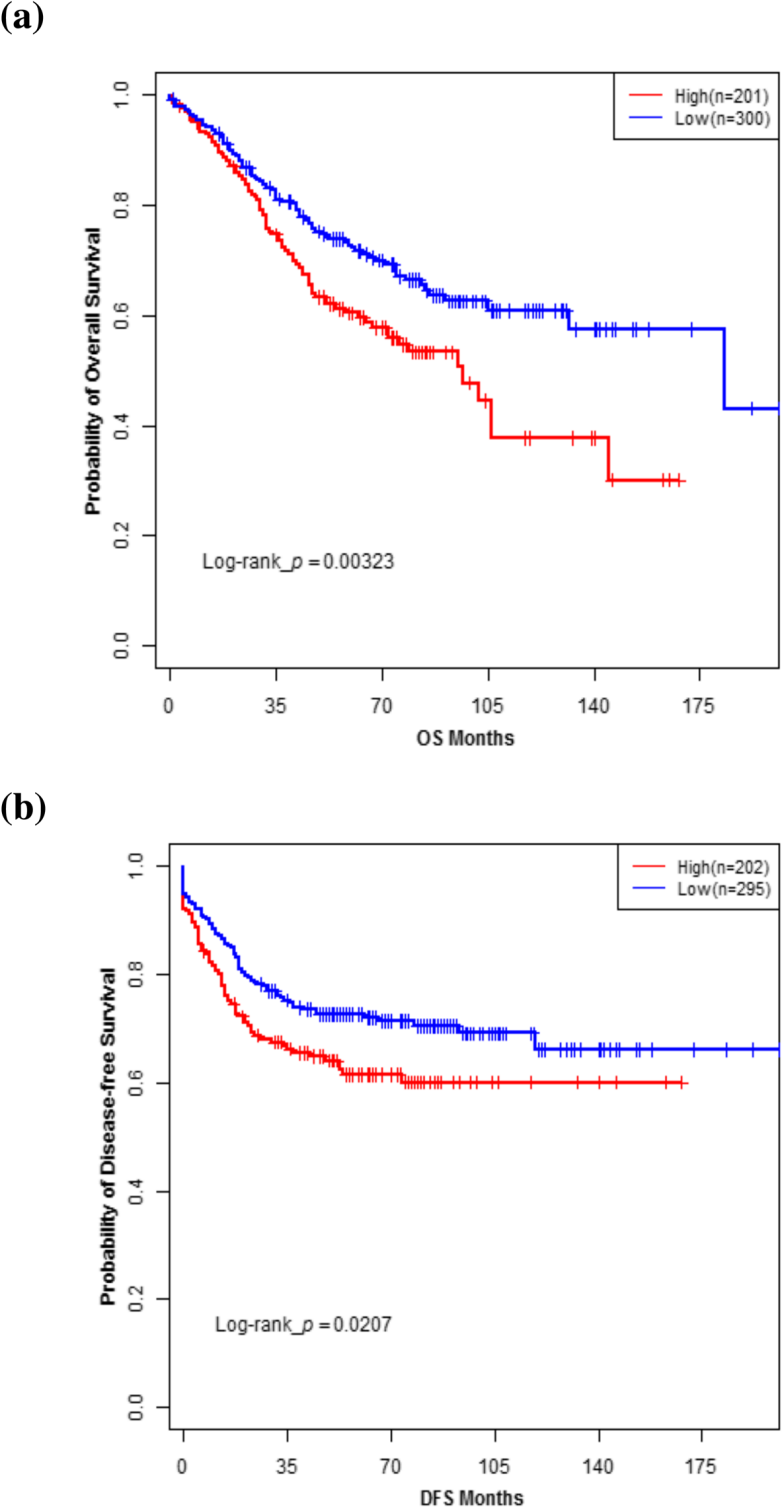
Kaplan-Meier survival analysis of colorectal cancer patients stratified by *SLC2A3* expression levels (Validation set). Overall survival (**a**) and disease-free survival (**b**) curve of patients in validation set with high versus low *SLC2A3* expression levels. P-values for significance of difference between high and low expression were calculated using the log-rank test

*SLC2A3* was significantly associated with both OS (*P* = 0.005) and DFS (*P* = 0.024). There was associations between the expression of *SLC2A1* with worse DFS (*P* = 0.015), but *SLC2A6* was not associated with worse OS (*P* = 0.940). The expression of *SLC2A7* was not provided. Patients in validation set were categorized into high vs. low *SLC2A3* expression according to the cut-off point. High expression led to worse OS (*P* = 0.003) and DFS (*P* = 0.021) (Fig. 2). In the multivariate Cox regression analysis, *SLC2A3* expression is associated with shorter OS (adjusted HR 1.50, 95% CI: 1.11–2.03; *P* = 0.009) and DFS (adjusted HR 1.38, 95% CI: 1.00–1.91; *P* = 0.048) after adjusting for clinical factors (age, AJCC stage for OS, AJCC stage for DFS) (Table 5).

**Table 5:** Univariate and multivariate Cox regression analyses of *SLC2A3* expression and the clinicopathological factors in the validation set (GSE39582)

	Overall survival		Disease-free survival	
	HR(95% CI of HR)	*P* value^a^	HR(95% CI of HR)	*P* value^a^
*SLC2A3* low	*1.00 (Reference)*		*1.00 (Reference)*	
UVA *SLC2A3* high	1.57 (1.16–2.12)	0.004	1.46 (1.06–2.01)	0.021
MVA *SLC2A3* high	1.50 (1.11–2.03)	0.009	1.38 (1.00–1.91)	0.048
Age < = 65	*1.00 (Reference)*			
Age > 65	1.39 (1.01–1.91)	0.043		
AJCC stage I, II	*1.00 (Reference)*		*1.00 (Reference)*	
AJCC stage III, IV	1.82 (1.34–2.46)	< 0.001	2.84 (2.03–3.98)	< 0.001

Abbreviations: *HR* hazard ratio, *CI* confidence interval, *UVA* univariate; MVA,multivariate

^a^Calculated using the Wald test

### Prognostic value of SLC2A1

**Fig. 3:**
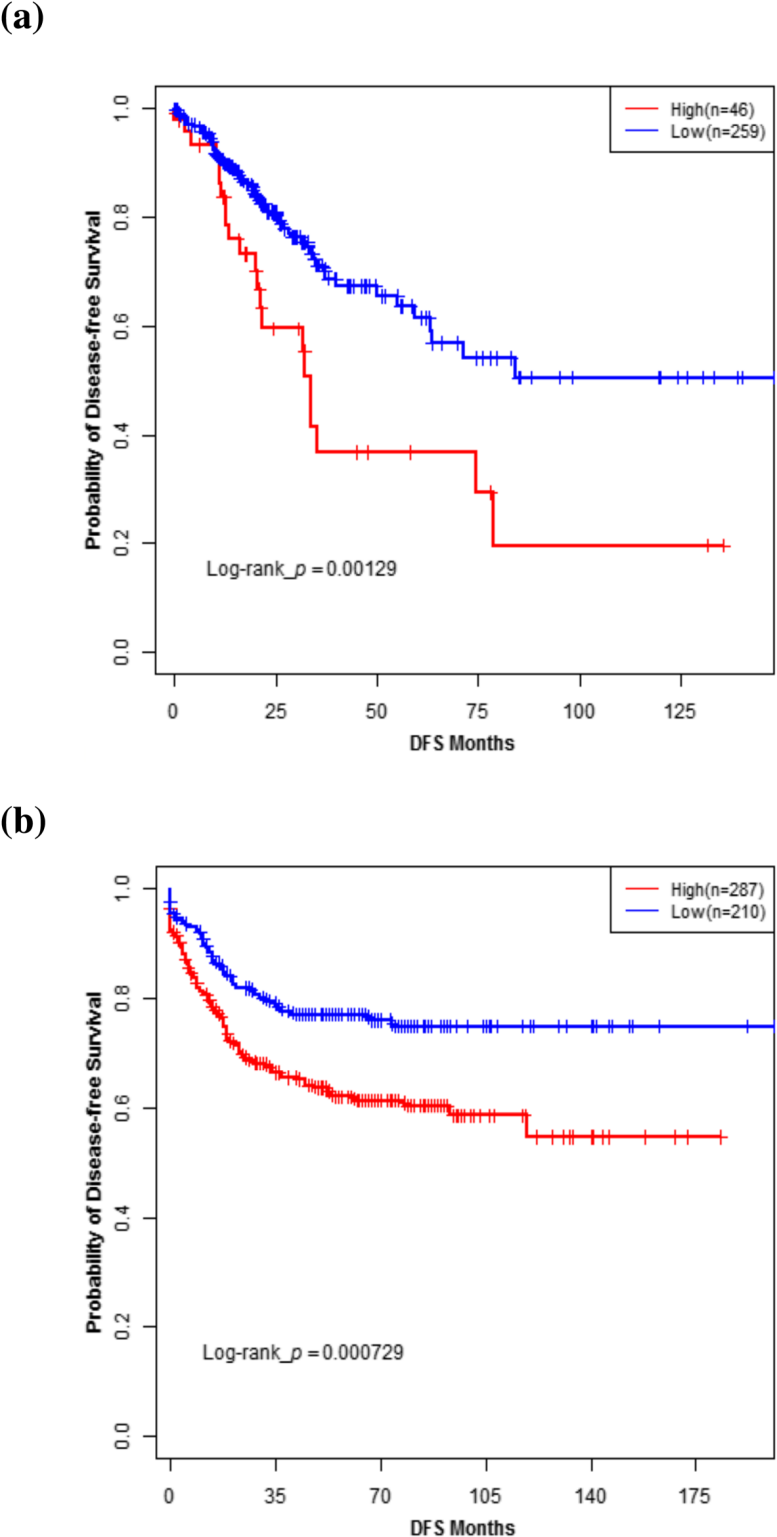
Kaplan-Meier survival analysis of colorectal cancer patients stratified by *SLC2A1* expression levels in Discovery set and Validation set. Disease-free survival curve of patients in Discovery set (**a**) and Validation Set (**b**) with high versus low *SLC2A1* expression levels. P-values for significance of difference between high and low expression were calculated using the log-rank test

Patients were categorized into high and low *SLC2A1* expression groups according to the cut-off value determined by Maxstat method. The high expression group had worse DFS in both Discovery set and Validation set (*P* = 0.001 and < 0.001, respectively) compared to the low expression group (Fig. 3). We explored the association between *SLC2A1* expression and DFS in a multivariate context of Discovery set and Validation set. In multivariate Cox regression analysis, after adjusting for AJCC TNM stage III and IV that were significantly associated with DFS, *SLC2A1* remained an independent poor prognostic factor for DFS in both Discovery set (adjusted HR 1.83, 95% CI: 1.12–3.01; *P* = 0.017) and Validation set (adjusted HR 1.65, 95% CI: 1.14–2.40; P = 0.009).

## Discussion

Most cancer cells favor glycolytic energy metabolism over mitochondrial metabolism and oxidative phosphorylation chain for energy production, even in the presence of oxygen (Warburg effect). This explains why malignant cells overexpress GLUT family proteins, which is a plasma membrane transport system. Glucose can be translocated into the cell only via GLUT proteins. Expression and subcellular distribution of GLUT proteins are regulated by different signaling molecules and pathways such as *PI3K*, *HIF*, *p53*, *Myc*, and *AMPK LBk1* [11, 12].

In the current study, we used the TCGA database to explore the clinical significance of SLC2 family genes in CRC. IHC staining of GLUT expression in cancer cells can be diverse which is a downside with IHC staining. This may be caused by incorrectly interpreted IHC stain results or different GLUT positive thresholds [9]. Messenger RNA-gene expression analysis from TCGA data is superior to IHC and may best predict cancer prognosis in TCGA data [13]. The current study is the first to report the expression of *SLC2A* genes in CRC using the TCGA database.

In the current study, age and AJCC TNM stage were associated with survival in CRC patient. As expected, in this study, age > 65 was linked with worse prognosis for CRC patients. This may be due to aging itself, or due to co-morbidities such as cardiovascular disease or postoperative complications [14]. Older patients are less likely to be treated with resectional surgery than younger patients and have poorer survival outcomes [15]. We included age > 65 as a factor for multivariate analysis because age > 65 is useful for prognostication. We found that AJCC stage III and IV, which has nodal metastasis or organ metastasis, was associated with OS and DFS in the TCGA database. AJCC TNM stage was established based on OS, and lymph node involvement in itself is considered to have a strong influence on OS and DFS in CRC [16]. In oral squamous cell carcinoma and papillary thyroid carcinoma, GLUT3 was known to be a prognostic marker for OS, and was associated with advanced cancer stage (AJCC TNM stage III and IV) which has nodal metastasis [8, 17]. In current study, we found high expression of *SLC2A3* was a prognostic factor for predicting OS and DFS in CRC and was not associated with AJCC TNM stage. Similar to current findings, GLUT3 is a significant marker of poor prognosis in laryngeal carcinoma, with no significant differences in nodal or distant metastasis between the GLUT3 negative and GLUT3 positive groups [18]. Since *SLC2A3* or GLUT3 genes are associated with prognosis in CRC as well as thyroid or laryngeal carcinoma, it would be worthwhile to investigate whether these cancers have similar GLUT-dependent metabolic pathways.

In the current study, we analyzed the mRNA expression values of all 14 *SLC2A* family genes and found that *SLC2A3* is independently associated with both OS and DFS after adjusting for age and AJCC TNM stage in CRC patients. Among the GLUT family, GLUT1 is associated with prognosis in several cancers, including CRC. GLUT3 has high affinity glucose uptake, similar to GLUT1 [19]. GLUT3 is overexpressed in human carcinomas including CRC [20]. Although GLUT1 and GLUT3 have many similarities, including expression pathways, the effects of GLUT3 on outcomes in several cancer varieties are not as well understood as GLUT1. This is the first report to study the effects of *SLC2A3* on CRC patients’ survival.

The following reasons may explain why the expression of *SLC2A3* affects CRC prognosis. GLUT3 is induced by hypoxia-inducible factor (HIF) formation in response to hypoxia in carcinomas [11]. HIF-1 promotes tumor metastasis into distant and more oxygenated tissue through the transcriptional activation of oncogenic growth factors such as transforming growth factor beta3, epidermal growth factor, and others [21]. Solid tumors with high hypoxia levels are more malignant, more likely to metastasize, and have a worse prognosis [11]. GLUT3 is also induced by Akt involved in the Warburg effect. In cancer cells, Akt increases expression of GLUT1 and GLUT3 by causing degradation of p53.It may reflect the activity of hypoxia independent oncogenic pathways [11, 19].

*SLC2A1* expression was associated with poor DFS but not with OS. This corresponds well with a study which reported that GLUT-1 expression is associated with poor DFS but not with OS in rectal cancer patients [9]. To further evaluate the association between the expression of *SLC2A1* and rectal cancer, we performed a subgroup analysis for rectal cancer patients using a univariate Cox regression analysis. Although we were unable to confirm our findings in the validation set due to a lack of primary site information, in the discovery set, *SLC2A1* expression in rectal cancer patients (*n* = 72) was significantly associated with DFS (HR 1.57, 95% CI: 1.04–2.38; *P* = 0.03). We also found no association between *SLC2A1* expression and OS (HR 1.54, 95% CI: 0.96–2.46; *P* = 0.07) in rectal cancer patients. Our findings were consistent with the findings of the previous study.

In addition to *SLC2A* family genes, the following 12 genes are known to be involved in glucose metabolism: *MTOR*, *RICTOR*, *HIF1A*, *MYC*, *PDK1*, *PDK2*, *PDK3*, *PDK4*, *PIK3R1*, *PKM*, *POU2F-1*, and *RPTOR* [12]. We conducted an analysis to evaluate if the expression of the genes was associated with survival outcomes of CRC. We found no association between the other 12 genes’ mRNA expression and survival outcomes of CRC in both discovery and validation sets (Additional file 1: Table S1). Based on these findings, we concluded that among the glucose metabolism regulating genes, only the *SLC2A3* gene is significantly associated with the survival outcomes of CRC.

There are several genes known to be associated with the prognosis of CRC: *BRAF*, *KRAS*, *HIF*, *TP53* and thymidylates synthase (TYMS) [22, 23]. The *BRAF* mutation generates an abnormality in the MEK/ERK signaling pathway in CRC [24] and has been reported to be associated with poor prognosis by many CRC studies. However, reports vary on its association with survival [25]. Mutation of *KRAS*, a proto-oncogene, activates *RAS* signaling pathways, but its association with CRC survival is not clear [24, 26]. In the current study, we analyzed the association of *BRAF* and *KRAS* expression level with survival, and found neither to be associated with OS or DFS in the discovery set (Additional file 1: Table S2, Table S3 and Additional file 2: Figure S1). Likewise, in the recent study conducted by authors’ group, the mutational status of *BRAF* or *KRAS* was not associated the prognosis of CRC [27]. For *HIF*, *TP53* and *TYMS*, there are studies which reported the expression of the genes are not related to the prognosis of OS of CRC [28–30]. In this study, we also found that *HIF*, *TP53* and *TYMS* were not associated with OS or DFS (Additional file 1: Table S4). The discrepancies among the studies regarding the prognostic value of these genes may result from differences in patient cohorts, available co-variates, or statistical methods.

The short observation period of the discovery set patients (31.4 months median follow-up period) is a limitation of our study. Secondly, the relationship between the expression of *SLC2A* mRNA and GLUT protein in CRC has not been confirmed. Although one study reported a close correlation between the expression of *SLC2A1*/*SLC2A3* mRNA and that of GLUT1/GLUT3 proteins in thyroid carcinoma [31], further studies are needed to investigate whether the expression of *SLC2A* mRNA correlates with expression of GLUT protein mRNA in CRC. Another limitation of our results is that mRNA gene expression value is not a readily available parameter, especially in clinical settings, due to the high cost of storage and processing of fresh tissue. Its application may become wider in the near future when cost is reduced and stable mRNA expression can be obtained through formalin-fixed paraffin-embedded tissue samples.

## Conclusions

In conclusion, upregulation of the *SLC2A3* gene is associated with decreased OS and DFS in CRC patients. *SLC2A3* gene expression analysis may be useful for predicting prognosis and survival of CRC patients.

## Additional files

Additional file 1: **Table S1.** Univariate cox regression analysis of glycolysis related genes for OS and DFS **Table S2.** Univariate and multivariate Cox regression analyses of *BRAF* mutation and the clinicopathological factors in the discovery set (TCGA COADREAD cohort) **Table S3.** Univariate and multivariate Cox regression analyses of *KRAS* mutation and the clinicopathological factors in the discovery set (TCGA COADREAD cohort) **Table S4.** Univariate Cox regression analysis of HIF, TP53, TYMS genes for OS and DFS in the discovery set (TCGA COADREAD cohort) (DOCX 25 kb)

Additional file 2: **Figure S1.** Kaplan-Meier Survival analysis for overall survival and disease-free survival in colorectal cancer patients according to mutational status in the Discovery set. Overall survival (a) and disease-free survival (b) according to *BRAF* mutation status. Overall survival (c) and disease-free survival (d) according to *KRAS* mutation status. **Figure S2.** R code used for statistical analysis. The datasets analyzed during the study are available in the following repositories: TCGA COADREAD : cBioportal for cancer genomics (http://www.cbioportal.org.). GSE39582: Gene Expression Omnibus (GEO)(https://www.ncbi.nlm.nih.gov/geo/.) (PDF 122 kb)

## Abbreviations

AJCC: American Joint Committee on Cancer
*BRAF*: v-Raf murine sarcoma viral oncogene homolog B1 genes
CI: Confidence interval
CRC: Colorectal cancer
DFS: Disease-free survival
GLUT: Glucose transporter
HR: Hazard ratio
IHC: immunohistochemical
*KRAS*: v-Ki-ras2 Kirsten rat sarcoma viral oncogene homolog
OS: Overall survival
*SLC2A*: Solute carrier 2A
TCGA: the cancer genome atlas
