# Supplementary material for: Upregulation of SLC2A3 gene and prognosis in colorectal carcinoma: analysis of TCGA data
Source: BMC Cancer. 2019 Apr 3;19:302. doi: 10.1186/s12885-019-5475-x (PMC6446261; doi:10.1186/s12885-019-5475-x)
Supplement: Supplementary file 1 — Table S1. Univariate cox regression analysis of glycolysis related genes for OS and DFS Table S2. Univariate and multivariate Cox regression analyses of BRAF mutation and the clinicopathological factors in the discovery set (TCGA COADREAD cohort) Table S3. Univariate and multivariate Cox regression analyses of KRAS mutation and the clinicopathological factors in the discovery set (TCGA COADREAD cohort) Table S4. Univariate Cox regression analysis of HIF, TP53, TYMS genes for OS and DFS in the discovery set (TCGA COADREAD cohort) (DOCX 25 kb) [file 12885_2019_5475_MOESM1_ESM.docx]

**Table S1** Univariate cox regression analysis of glycolysis related genes for OS and DFS

| **Gene**^a^ | **Discovery Set** | | | | **Validation Set** | | | | | |
| --- | --- | --- | --- | --- | --- | --- | --- | --- | --- | --- |
|  | **OS** | | **DFS** | | **OS** | | **DFS** | | |  |
|  | **HR (95% CI)** | **P value**^b^ | **HR (95% CI)** | **P value**^b^ | **HR (95% CI)** | **P value**^b^ | | **HR (95% CI)** | **P value**^b^ | |
| *MTOR* | 0.89(0.73-1.09) | 0.270 | 0.97(0.78-1.19) | 0.739 | 0.95(0.81-1.1) | 0.463 | | 0.93(0.79-1.1) | 0.390 | |
| *RICTOR* | 1.29(1.01-1.63) | 0.039 | 1.1(0.88-1.37) | 0.399 | 1.11(0.96-1.29) | 0.177 | | 1.09(0.93-1.28) | 0.286 | |
| *HIF1A* | 0.9(0.71-1.13) | 0.368 | 0.96(0.77-1.21) | 0.753 | 1(0.86-1.17) | 0.997 | | 0.99(0.84-1.16) | 0.872 | |
| *MYC* | 0.8(0.64-1.02) | 0.067 | 0.87(0.69-1.09) | 0.213 | 0.84(0.73-0.97) | 0.021 | | 0.85(0.73-0.99) | 0.040 | |
| *PDK1* | 1.02(0.82-1.27) | 0.865 | 0.94(0.77-1.16) | 0.580 | 0.97(0.84-1.12) | 0.686 | | 0.92(0.78-1.08) | 0.289 | |
| *PDK2* | 1.06(0.83-1.36) | 0.627 | 0.97(0.76-1.23) | 0.777 | 0.94(0.81-1.09) | 0.409 | | 1(0.86-1.18) | 0.952 | |
| *PDK3* | 0.94(0.74-1.18) | 0.595 | 0.98(0.79-1.23) | 0.868 | 0.96(0.83-1.11) | 0.572 | | 1.05(0.9-1.23) | 0.528 | |
| *PDK4* | 0.93(0.74-1.17) | 0.547 | 0.96(0.78-1.18) | 0.699 | 1.28(1.12-1.46) | <0.001 | | 1.29(1.13-1.49) | <0.001 | |
| *PIK3R1* | 0.94(0.74-1.19) | 0.609 | 0.87(0.69-1.1) | 0.236 | 0.85(0.73-0.99) | 0.035 | | 0.88(0.75-1.04) | 0.129 | |
| *PKM* | 1.13(0.91-1.41) | 0.267 | 1.11(0.89-1.39) | 0.341 | 1.04(0.89-1.21) | 0.62 | | 1.01(0.86-1.19) | 0.903 | |
| *POU2F1* | 1.47(1.18-1.83) | 0.001 | 1.33(1.07-1.67) | 0.012 | 0.93(0.79-1.08) | 0.331 | | 0.85(0.73-1) | 0.057 | |
| *RPTOR* | 1(0.8-1.25) | 0.999 | 1(0.81-1.24) | 0.982 | 0.94(0.81-1.1) | 0.452 | | 1.02(0.87-1.19) | 0.820 | |

Abbreviations: OS overall survival, DFS disease-free survival, HR hazard ratio, CI confidence interval

^a^Unit of measure is log2 of gene expression intensity.

^b^Calculated using the Wald test.

**Table S2** Univariate and multivariate Cox regression analyses of *BRAF* mutation and the clinicopathological factors

|  |  | |  |  | | |  | |
| --- | --- | --- | --- | --- | --- | --- | --- | --- |
|  | | **Overall survival** | | | | **Disease-free survival** | | |
|  | | **HR(95% CI of HR)** | | | **P value**^a^ | **HR(95% CI of HR)** | | **P value**^a^ |
| ***BRAF* wildtype** | | *1.00 (Reference)* | | |  | *1.00 (Reference)* | |  |
| UVA *BRAF* mutant | | 1.26(0.69-2.28) | | | 0.452 | 0.81(0.4-1.61) | | 0.544 |
| MVA *BRAF* mutant | | 1.11(0.6-2.05) | | | 0.734 | 0.84(0.42-1.67) | | 0.614 |
| **Age <= 65** | | *1.00 (Reference)* | | |  |  | |  |
| Age >65 | | 2.35(1.41-3.9) | | | <0.001 |  | |  |
| **AJCC stage I, II** | | *1.00 (Reference)* | | |  |  | |  |
| AJCC stage III, IV | | 3.13(1.95-5.04) | | | <0.001 | 2.64(1.69-4.13) | | <0.001 |

Abbreviations: BRAF v-Raf murine sarcoma viral oncogene homolog B1 genes, HR hazard ratio, CI confidence interval, UVA univariate, MVA multivariate, AJCC American Joint Committee on Cancer

^a^Calculated using the Wald test.

**Table S3** Univariate and multivariate Cox regression analyses of *KRAS* mutation and the clinicopathological factors

|  |  |  |  |  |
| --- | --- | --- | --- | --- |
|  | **Overall survival** | | **Disease-free survival** | |
|  | **HR(95% CI of HR)** | **P value**^a^ | **HR(95% CI of HR)** | **P value**^a^ |
| ***KRAS* wildtype** | *1.00 (Reference)* |  | *1.00 (Reference)* |  |
| UVA *KRAS* mutant | 0.80(0.5-1.26) | 0.328 | 1.07(0.69-1.65) | 0.760 |
| MVA *KRAS* mutant | 0.83(0.52-1.32) | 0.437 | 1.22(0.79-1.89) | 0.371 |
| **Age <= 65** | *1.00 (Reference)* |  |  |  |
| Age >65 | 2.45(1.48-4.04) | <0.001 |  |  |
| **AJCC stage I, II** | *1.00 (Reference)* |  |  |  |
| AJCC stage III, IV | 3.05(1.89-4.92) | <0.001 | 2.72(1.73-4.27) | <0.001 |

Abbreviations: KRAS v-Ki-ras2 Kirsten rat sarcoma viral oncogene homolog, HR hazard ratio, CI confidence interval, UVA univariate, MVA multivariate, AJCC American Joint Committee on Cancer

^a^Calculated using the Wald test.

**Table S4** Univariate cox regression analysis of *HIF*, *TP53*, *TYMS* genes for OS and DFS

| **Gene**^a^ | **Discovery Set** | | | | **Validation Set** | | | | | |
| --- | --- | --- | --- | --- | --- | --- | --- | --- | --- | --- |
|  | **OS** | | **DFS** | | **OS** | | **DFS** | | |  |
|  | **HR (95% CI)** | **P value**^b^ | **HR (95% CI)** | **P value**^b^ | **HR (95% CI)** | **P value**^b^ | | **HR (95% CI)** | **P value**^b^ | |
| *HIF1A* | 0.9(0.71-1.13) | 0.368 | 0.96(0.77-1.21) | 0.753 | 1(0.86-1.17) | 0.997 | | 0.99(0.84-1.16) | 0.872 | |
| *TP53* | 0.92(0.74-1.15) | 0.469 | 0.85(0.7-1.04) | 0.112 | 0.96(0.83-1.11) | 0.555 | | 0.94(0.81-1.1) | 0.429 | |
| *TYMS* | 0.9(0.72-1.13) | 0.384 | 0.85(0.69-1.06) | 0.154 | 0.88(0.76-1.03) | 0.114 | | 0.76(0.65-0.89) | 0.001 | |

Abbreviations: OS overall survival, DFS disease-free survival, HR hazard ratio, CI confidence interval

^a^Unit of measure is log2 of gene expression intensity.

^b^Calculated using the Wald test.
