# Supplementary material for: Upregulation of SLC2A3 gene and prognosis in colorectal carcinoma: analysis of TCGA data
Source: BMC Cancer. 2019 Apr 3;19:302. doi: 10.1186/s12885-019-5475-x (PMC6446261; doi:10.1186/s12885-019-5475-x)
Supplement: Supplementary file 2 — Figure S1. Kaplan-Meier Survival analysis for overall survival and disease-free survival in colorectal cancer patients according to mutational status in the Discovery set. Overall survival (a) and disease-free survival (b) according to BRAF mutation status. Overall survival (c) and disease-free survival (d) according to KRAS mutation status. Figure S2. R code used for statistical analysis. The datasets analyzed during the study are available in the following repositories: TCGA COADREAD : cBioportal for cancer genomics (http://www.cbioportal.org.). GSE39582: Gene Expression Omnibus (GEO)(https://www.ncbi.nlm.nih.gov/geo/.) (PDF 122 kb) [file 12885_2019_5475_MOESM2_ESM.pdf]

# Figure S1

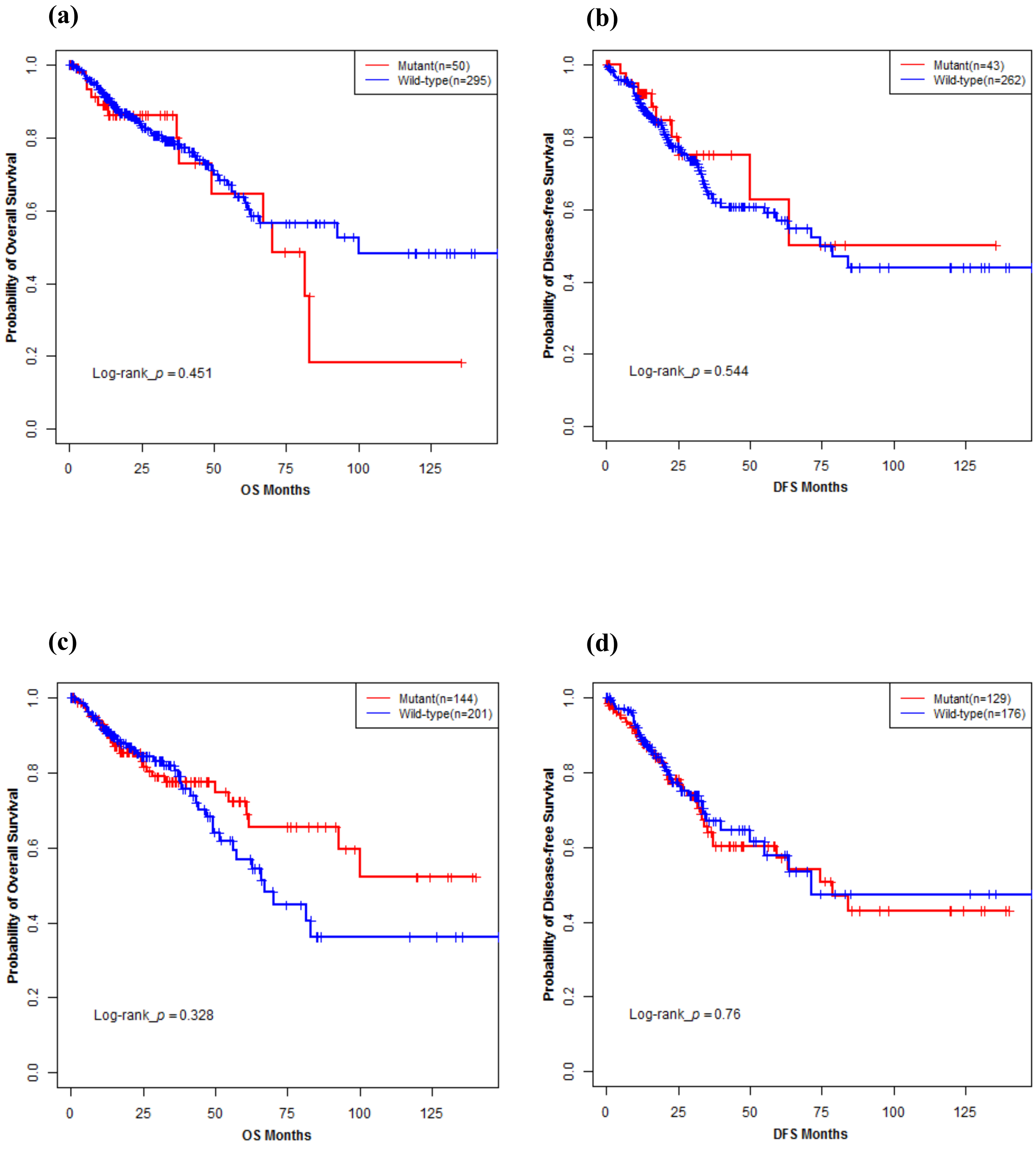
(a)
Mutant(n=50)
Wild-type(n=295)
Probability of Overall Survival
Log-rank_p = 0.451
OS Months
(b)
Mutant(n=43)
Wild-type(n=262)
Probability of Disease-free Survival
Log-rank_p = 0.544
DFS Months
(c)
Mutant(n=144)
Wild-type(n=201)
Probability of Overall Survival
Log-rank_p = 0.328
OS Months
(d)
Mutant(n=129)
Wild-type(n=176)
Probability of Disease-free Survival
Log-rank_p = 0.76
DFS Months

# Figure S2

```
######################################################################################
rm(list=ls())
library(survival)
library(survminer)
library(maxstat)

ds1<- read.csv("discoveryset.csv",sep=",",header=T) ## for validation, validation set was used

## Survival information
# for OS analysis (for DFS analysis, DFS_MONTHS and DFS_STATUS were used)
ds1$time<-ds1$OS_MONTHS
ds1$status<-ds1$OS_STATUS

## The optimal cut-off points for SLC2A3 expression
optimalCut <- maxstat.test(Surv(time, status) ~ SLC2A3,data=ds1, smethod="LogRank",
pmethod="HL")
ds1$SLC2A3_high<-as.factor(ifelse(ds1$SLC2A3<=optimalCut$estimate,0,1))

## Chi-square test
ds1$Age2<-as.factor(ifelse(ds1$Age<=65,0,1))
ds1$TNM.stage2<-as.factor(ifelse(ds1$TNM.stage%in%c("stage I","stage II"),0,1))
chisq.test(table(ds1$Age2, ds1$SLC2A3_high))$p.value
chisq.test(table(ds1$sex, ds1$SLC2A3_high))$p.value
chisq.test(table(ds1$TNM.stage2, ds1$SLC2A3_high))$p.value
chisq.test(table(ds1$MSI, ds1$SLC2A3_high))$p.value
chisq.test(table(ds1$BRAF, ds1$SLC2A3_high))$p.value
chisq.test(table(ds1$KRAS, ds1$SLC2A3_high))$p.value

## Kaplan-Meier plot and log-rank test
library(ggkm)
```

```
fit=survfit(Surv(time,status)~SLC2A3_high,data=ds1)
ggkm(fit,pval=T)


## Univariate and multivariate Cox proportional-hazards model(with Wald test)
coxph(Surv(as.numeric(time), as.numeric(status))~SLC2A3_high,data=ds1)
coxph(Surv(as.numeric(time), as.numeric(status))~TNM.stage2+age2+SLC2A3_high,data=ds1)
#############################################################################
```
